# Navigating complexity: globalization narratives in China and the West

**DOI:** 10.1007/s42533-022-00113-2

**Published:** 2023-01-10

**Authors:** Anthea Roberts, Nicolas Lamp

**Affiliations:** 1 School of Regulation and Global Governance (RegNet), College of Asia & the Pacific, Australian National University, Canberra, Australia; 2 Faculty of Law, Queen’s University, Kingston, Canada

**Keywords:** Globalization, Narratives, China, United States, Complexity, Multi-perspective approach

## Abstract

Relations between China and the West appear to be caught in a downward spiral. In the West, there is a widespread perception that China has unduly benefited from economic globalization, while in China, there appears to be increasing concern that the West is seeking to contain China’s rise. In this essay, we argue that the picture is more complex. We first discuss the highly varied ways in which China appears in Western narratives about economic globalization. We then sketch our understanding of how different narratives about globalization are playing out in China. Our approach highlights the diversity of perspectives within and between the West and China. How countries, companies, and individuals navigate this complexity depends not just on the rise and fall of narratives within the West and China, but also on how these narratives intersect and interact with each other.

## Introduction

Complex issues look different from different perspectives. In *Six Faces of Globalization: Who Wins, Who Loses, and Why It Matters*, we track the main narratives that have dominated Western debates about economic globalization in recent years. We argue that no one view holds the whole truth. Competing narratives identify different winners and losers of economic globalization while advancing different claims about whether these wins and losses are good or bad. However, a common theme, particularly since 2016, has been the growing pushback against the upbeat establishment account of economic globalization from narratives that focus on issues such the increasing class divide in Western societies, the West’s relative loss of economic power, and skyrocketing carbon emissions. In the West, globalization’s discontents have been growing in number and strength.

But the backlash against economic globalization in Western countries is not the whole story. Perspectives on globalization in countries outside the West differ significantly from the views that dominate Western media and political debates. As the Singaporean public intellectual and former diplomat Kishore Mahbubani notes, “for the majority of us, the past three decades—1990 to 2020—have been the best in human history,” as hundreds of millions lifted themselves out of poverty, and living standards soared across much of the developing world (Mahbubani 2018). Parag Khanna, the author of the book *The Future Is Asian*, concurs: “Western populist politics from Brexit to Trump haven’t infected Asia.... Rather than being backward-looking, navel-gazing, and pessimistic, billions of Asians are forward-looking, outward-orientated, and optimistic” (Khanna 2019). Globalization continues to have many supporters around the globe, particularly in Asia.

Where does China fit into this story? In this essay, we explore two aspects of this question: what role does China play in the Western narratives, and what role do these or other narratives play in China? We first explore the multiple ways China appears in different Western narratives—as a poster child, a villain, a scapegoat, a threat, and an indispensable nation. We then sketch our understanding of how different narratives about globalization are playing out in China, from the embrace of free trade as a driver of prosperity to increased concerns about the economic and security implications of rising geoeconomic tensions to a turn toward common prosperity instead of simple economic growth.

Our multi-narrative analysis highlights the diversity of perspectives not just within but also between the West and China—an approach that psychologists have found to be useful in understanding complex issues more holistically and in identifying potential pathways forward.^1^ How countries, companies, and individuals navigate these complexities depends not just on the rise and fall of narratives within the West and China, but also on how these narratives intersect and interact with each other on the international plane and, in turn, influence domestic politics and policies in an iterative and recursive manner.^2^ Narratives in the West about China can affect narratives in China about the West, which can in turn affect narratives in the West and so on. As relations between the West and China become more fraught, understanding this interplay becomes ever more important.

## China’s role in western narratives about globalization

The relationship between the West and China appears to be caught in a downward spiral. The trade war initiated by former US President Trump has morphed into simmering hostility under the Biden administration. In quarrels over politically sensitive questions, such as an international inquiry into the origins of the COVID-19 pandemic or relations with Taiwan, China has at times resorted to trade restrictions that its trading partners (such as Australia and the European Union) decry as economic coercion, prompting the lodging of legal challenges in the World Trade Organization and the development of anti-coercion instruments. The Chinese government’s decision not to condemn Russia’s invasion of Ukraine has further deepened estrangement between the two sides.

While an antagonistic view of the relationship has come to dominate public debates in the West, especially in the United States, just below the surface there are a variety of Western narratives that depict China’s role in the global economy in widely varying ways. Just as there is no one view of economic globalization, so too there is no one view of China and its role in the process. Examining these competing narratives not only provides a nuanced picture of Western debates but also highlights the contradictions and trade-offs that the West must navigate as it reassesses its relationship with China.

### The establishment position: China as a poster child

Not long ago, the perhaps dominant view of China in the West was that it was a poster child for economic globalization’s successes. After all, here was a country that had managed to fulfill the aspiration shared by every “developing country” since that concept was first coined in the period of decolonization, namely, to follow the path trodden by today’s “developed countries” while at the same time contributing to the economic growth of its trading partners.

China stuck to that economic development path almost to a tee: it first attracted labor-intensive manufacturing industries, then moved up the value chain by acquiring advanced technologies, and finally evolved from imitator to innovator to attain technological leadership in important sectors, just as the United States and Germany had done in the nineteenth and early twentieth centuries. In the process, China created the conditions for hundreds of millions of its citizens to lift themselves out of destitution in just a few short decades—a reduction of poverty on a scale and speed unparalleled in human history. Even as globalization has started to lose its luster in the West, the proponents of what we call the pro-globalization “establishment” narrative keep pointing to this historic achievement as a knockout argument for its benefits.

However, China not only benefited its own citizens by opening up to the global economy: its manufacturing prowess and its status as the largest and fastest-growing market in the world in many industries also benefited producers, service providers, consumers, and entrepreneurs all over the world. The prices of manufactured goods, from fridges to solar panels to clothing, have fallen dramatically as hundreds of millions of Chinese workers have joined the global labor force, producing large savings for consumers and allowing businesses to source cheap inputs and products, boosting their profits. Many multinational companies also generate a significant share of their global revenues from their businesses in China, supporting employment and R&D in their home countries. Technological collaboration has led to scientific advances and business innovation. On this view, China’s integration into the world economy produced an economic bonanza that showcased the potential of globalization to make (almost) everyone better off.

### The right-wing populist view: China as a villain

In the 2016 US presidential election, Donald Trump garnered support among US voters by painting China’s role in the global economy in starkly different terms. In his telling, China had achieved its astounding economic success not through hard work, ingenuity, and sacrifice, but by cheating its way around international trade rules. By using export subsidies to prop up its own companies, engaging in theft of intellectual property from Western companies, and undercutting labor and environmental standards, China had been able to “steal” the jobs of hard-working American manufacturing workers, leaving behind “rusted out factories” and devastated communities marked by unemployment and despair. In this view, Chinese workers had won at the expense of American workers.

This right-wing populist view, which continues to resonate among many American voters, discounts the benefits of trade with China, such as access to cheap products and to China’s large domestic market, and instead highlights the costs, which it measures first and foremost in terms of the decline of US manufacturing employment and the social ills that have followed in its wake. Proponents of this narrative argue for reshoring manufacturing in the hope of reviving communities in America’s rust belt so that they, rather than Chinese workers, may flourish again. In this view, China has taken advantage of America and left it weak; bringing back jobs in coal mines, steel smelters, and auto plants is vital to rebuilding the country’s industrial strength and making America great again.

### Left-wing and corporate power concerns: China as a scapegoat

Many on the political left share concerns about the effects of China’s economic practices on US manufacturing employment, but there is also another prominent theme in left-wing narratives: the charge that those on the political right use China as a scapegoat for problems that are in large part the product of domestic policies within Western countries.

On this view, blaming China for the malaise of the middle and working classes in developed economies obscures the role that domestic policy failures have played in widening the gap between the rich and the poor in many Western countries. From underinvestment in schools and infrastructure to restrictive zoning laws that drive up the cost of housing, from anti-union legislation to regressive tax codes, it is largely domestic policies that rig the economy in favor of an entrenched elite.

A key piece of evidence for this narrative is the fact that the socio-economic impact of trade with China has been highly uneven across Western economies. Countries with active labor market policies and developed welfare states have fared much better than those with lax social safety nets and few union protections: they have lower inequality, healthier populations, and less polarized electorates. Left-wing populists view the anti-China rhetoric in many domestic debates as a way of distracting from these domestic failures and demonizing an external “other” to cover class divisions occurring within these countries.

Some on the left also point to the complicity of Western corporations in China’s supposed misdeeds. After all, no one forced these companies to offshore their production to China or to source inputs from shady suppliers with questionable commitments to labor and environmental standards. Again, they argue that the culprit for the West’s problems can be found closer to home: in governments that negotiate corporate-friendly trade deals and in corporate cultures and governance structures that legitimize the offshoring of jobs while CEOs and shareholders reap billions. On this view, complaints against China often reflect an attempt to shift the blame away from ill-advised domestic policies and unscrupulous corporate actors.

### The geoeconomic perspective: China as a threat

For all the economic establishment’s crowing over globalization’s success in reducing absolute poverty, it cannot deny that China’s integration into the global economy has failed to achieve another aspiration, namely, to transform China into a more democratic and pluralistic society. In other Asian powers, such as Japan and South Korea, successful capitalist development went along with a degree of political convergence with the West. Not so in China, which has instead charted its own course ideologically in a way that has contributed to growing geostrategic rivalry with the West and has undermined the assumption that engagement would lead to democratization.

Strategic distrust is at the heart of this fourth view of China’s role in a globalized world: a geoeconomic perspective that sees an emboldened and increasingly capable China as an economic and security threat to the West. On this view, the deep economic ties between China and the West are a source of vulnerability rather than opportunity. Instead of increasing the prospects of peace and prosperity, economic interdependence enabled China to close the gap on the West economically, technologically, and militarily while multiplying avenues for coercion, espionage, and sabotage. In absolute terms, both China and America may have benefited from economic globalization. In relative terms, however, economic integration has helped China catch up to America in a way that now raises deep economic and security concerns in Washington, DC and other Western capitals.

Instead of lamenting the offshoring of manufacturing jobs because of its effects on working class communities, the geoeconomic narrative focuses on the US–China battle for technological supremacy, with a particular focus on AI, quantum computing, and 5G telecommunications. It reasserts the importance of sovereign capabilities and supply chain security and resilience. As tensions between China and the West mount, so too do calls for Western companies to reduce their reliance on China and to decouple in various ways, first and foremost when it comes to critical technologies and critical goods. While the establishment narrative believed that trade would lead to peace, the geoeconomic narrative sees trust and peace as preconditions for trade, at least in areas in which being dependent on the trading partner would produce significant vulnerabilities. These concerns are leading to increased calls for reshoring and ally-shoring, as well as a revival of industrial policy.

### Confronting global threats: China as an indispensable nation

For a fifth narrative, all complaints that the West may have about China must not obscure an inescapable reality: the gravest threat that humanity faces is climate change, and this threat cannot possibly be tackled if China is not on board. China is not only the largest emitter of greenhouse gases; it also plays a leading role in the production and deployment of renewable energy equipment. On this view, it may well be that China has not always played by the Western rules of the game, and it may also be that its security interests are not aligned with those of the West, but these are of secondary importance compared to the existential challenge of tackling the climate crisis.

For this narrative, China is an indispensable nation, and prioritizing cooperation with China is essential. Instead of falling into the trap of us-versus-them thinking, this narrative argues that China and the West must see themselves as engaged in a common fight to preserve a habitable planet. Only by working together can we effectively tackle global threats. The same logic applies to the fight against pandemics. Without the early scientific collaboration that took place after the discovery of the novel coronavirus and that facilitated the development of tests and vaccines, the COVID-19 pandemic would have claimed many more lives. On this view, we need more, not less, scientific and technological engagement between the West and China and more, not less, global cooperation.

## How different narratives play out in China

The role that China plays in Western narratives is important, but so too is the role that these and other narratives play in China. Although the importance of both questions is equal, our ability to speak to them is not. As Western scholars who are deeply immersed in Western debates about globalization, we can speak confidently on the first question but only tentatively on the second. This asymmetry is true for many Western scholars—few of whom have lived in China or have Chinese language skills. This lopsidedness is something that will need to be rebalanced as global power dynamics shift.

We are also conscious that narratives operate differently in China than in the West, with the Chinese Communist Party and President Xi Jinping playing a much greater role in shaping narratives—both directly through their pronouncements and campaigns and indirectly through censorship and other limits on free speech—than governments do in the West. That does not mean that there is a single coherent narrative about globalization in China; instead, the Party may keep several narratives in tension to maximize its room for maneuver. But it does mean that those narratives do not emerge from and clash in open debates in the same way as they do in the West. It also means that some nonmainstream narratives are suppressed, while others percolate openly on social media but tend not to make the official Chinese newspapers.

With those caveats in mind, what have we gleaned from our reading of Chinese materials and our discussions with Chinese scholars and experts about how these narratives play out in China? As in the West, there is no monolithic view of economic globalization in China. Instead, we observe multiple narratives at play, as well as the relative rise and fall of different narratives over time.

### The establishment view with Chinese characteristics: Win–Win globalization

In some respects, China has taken on the mantle of economic globalization’s main defender, embracing a Chinese version of the establishment narrative just as it was falling out of favor in the West. A signature moment for this narrative occurred in 2017, when President Xi took to the stage at the World Economic Forum in Davos to stand proudly against the rising tide of anti-globalization sentiment in the West, declaring that “We must remain committed to developing global free trade and investment, promote trade and investment liberalization and facilitation through opening-up and say no to protectionism” (Xi 2017a). This view encapsulates the win–win narrative about free trade according to which economic globalization is a positive force that has “powered global growth and facilitated movement of goods and capital, advances in science, technology and civilization, and interactions among peoples” (Xi 2017a). (The narrative glosses over areas in which China has not globalized, which range from strict capital controls to limits on access to its domestic market in certain areas to the Great Firewall around its internet.)

This view acknowledges that economic globalization has played a crucial role in allowing China, along with many other Asian nations, to transform their economies and to boost living standards at a remarkable pace. The offshoring of manufacturing may have led to the decline of America’s rust belt, but it also fueled the development of a rising middle class in China, resulting in gleaming new cities and unprecedented prosperity for hundreds of millions. These enviable results arise not just from the opportunities presented by economic globalization, but from hard work by ordinary Chinese people and from good management by the country’s leadership in opening up gradually to the global economy while successfully navigating its whirlpools and waves. As a result, according to President Xi, China has “stood up, grown rich, and is becoming strong.” No longer the sick man of Asia, the “Chinese nation, with an entirely new posture, now stands tall and firm” (Xi 2017b, 2019a).

Given this positive framing, it is not surprising that the classic neocolonial narrative—which views economic globalization as a form of exploitation of the Global South by the transnational capitalist class that comes predominantly from the Global North—is largely absent in China, even though it has historically shaped attitudes towards globalization in other major developing countries, such as Brazil, India, and South Africa. Moreover, in the Party’s and Xi’s telling, what is good for China is also good for the world—a true win–win scenario. “China’s development is an opportunity for the world,” Xi explains, as “China has not only benefited from economic globalization but also contributed to it” (Xi 2017a). For many years, China’s growth has lent momentum to the world economy, which became particularly important when it helped to offset the recessions in the West that resulted from the Global Financial Crisis.

Not only has China been the engine of global growth, but President Xi suggests that China’s experience can provide a model for other countries seeking to follow China’s development trajectory. “We will open our arms to the people of other countries and welcome them aboard the express train of China’s development,” Xi has declared, suggesting that China would not be jealous of the achievements of other countries, unlike certain other unnamed countries (read: the United States) whom Xi implies have reacted jealously to China’s rise (Xi 2017a). China is able to supercharge the growth of other developing countries by providing global goods, such as fast and cheap infrastructure investment, making it a “leading dragon” in the tradition of Kaname Akamatsu’s “flying geese” paradigm of development.^3^ A concrete manifestation of this outward looking agenda is the Belt and Road Initiative, which aims to bring infrastructure development and deeper trade and investment ties to Chinese trading partners from Asia to Europe to Africa.

The Belt and Road Initiative is a key building block of President Xi’s vision of a “human community with a shared future,” which embraces some features of the liberal international order created after the Second World War, such as the sovereign equality of states and noninterference in internal affairs, while also supporting changes to that order (a point we explore in the next section) (Xi 2019a, b, c). Instead of being a predatory state engaged in debt trap diplomacy, as China is sometimes portrayed in the Western media, Chinese officials and media present their country as encouraging global development through foreign investment, infrastructure building, and (at times) debt forgiveness.

### The anti-establishment view: against western hegemony

In a different—and decidedly more negative—vein, the Chinese leadership, along with Russia, sometimes adopts an anti-hegemony narrative which charges that the West is trying to use globalization to universalize its model of liberal democracy and market-led capitalism. This narrative paints the West as hypocritical and hegemonic. On this view, Western countries are hypocritical because they wrote the global rules and expect other countries to follow those rules while often exempting themselves from the same standards. And Western countries are hegemonic in that they use their power to promote a one-size-fits-all model of political, social, and economic organization. Proponents of this critical narrative insist that different models must be respected and that multipolarity, not hegemony, must be the global organizing principle.

The Global Financial Crisis led to a significant loss of prestige in China for the West’s economic model and boosted the Chinese leadership’s resolve to chart its own path. As Wang Wen, a Chinese Communist Party member and a former chief opinion editor of *The Global Times*, explains:

[I]n contrast to my university days, the tone of Chinese academic research on the United States has shifted markedly. Chinese government officials used to consult me on the benefits of American capital markets and other economic concepts. Now I am called upon to discuss U.S. cautionary tales, such as the factors that led to the financial crisis. We once sought to learn from U.S. successes; now we study its mistakes so that we can avoid them (Wang 2022).

But the narrative against Western hegemony goes well beyond a rejection of the US model. In a joint declaration adopted in 2016, Russia and China explicitly take aim at the Western—and in particular the US—practice of using unilateral sanctions and the extraterritorial application of domestic laws to shape international developments. For Russia and China, this practice represents an application of “double standards” and the “imposition by some States of their will on other States,” as well as a violation of the principle of non-intervention (Anderson 2016). In another joint statement adopted in February 2022—just before Russia’s attack against Ukraine—the two governments lament the attempts by “certain States … to impose their own ‘democratic standards’ on other countries [and] to monopolize the right to assess the level of compliance with democratic criteria,” among other “attempts at hegemony” (Kremlin.ru. 2022).

According to this narrative, economic globalization may be good, but Western hegemony is bad. Every state should be permitted to chart its own path to development, Chinese officials regularly declare. As President Xi states: “No country should view its own development path as the only viable one, still less should it impose its own development path on others” (Xi 2017a). In the face of escalating pressure from the West, Chinese officials have insisted that China will not compromise its “core interests” or its model of development and will “struggle” against those who seek to contain its rise (Zhou and Zheng 2019). China suffered the “Century of Humiliation” at the hands of Western oppressors in the past, they remind national and international audiences. Now that China has grown strong, it will not allow itself to be the victim of such humiliation or containment again.

The perceived need to take a stand against Western hegemony has become more pronounced in recent years as a series of crises have rendered the relationship between the West and China more hostile. Chinese officials and commentators charge that Western countries, instead of celebrating China’s hard-earned economic success, are engaging in Cold War thinking and have invented a “China threat theory” to justify taking geoeconomic measures to contain China’s rise.^4^ These actions have led to calls within China to decouple from the West economically and technologically in everything from the internet and information flows (in which decoupling already exists to a large degree) to payment systems and technology (in which decoupling is suggested as a means of reducing Western leverage and Chinese vulnerability). On this view, China’s advances have pricked Western insecurities, leading to a backlash that China must now navigate.

Interdependence may be a source of economic gains, but it can also be a source of vulnerability. As hostility increases, those vulnerabilities have been front of mind for many in China. In increasingly securitized debates about interdependence, Xi has endorsed a broad notion of “national security” or “big security” that encompasses “economic security,” including “the security of important industries and key areas that are related to the lifeline of the national economy.”^5^ He has increasingly invoked the importance of zìlìgēngshēng (often translated as self-reliance and self-sufficiency) with respect to core technologies and emphasized the need for indigenization of technologies (e.g., Made in China 2025) to prevent the United States and other Western countries from exercising a chokehold over key items, such as semiconductors. “Advanced technology is the sharp weapon of the modern state,” observes Xi. “We must make a big effort in key fields and areas where there is a stranglehold.”^6^

Although some prominent Chinese thinkers view potential decoupling as “dangerous” and a “disaster for both China and the United States and the whole world,” an increasing number of elite Chinese thinkers have come to accept it as inevitable, particularly given US geoeconomic actions with respect to key Chinese technology firms such as Huawei, ZTE, Semiconductor Manufacturing International Corporation (SMIC), and Hikvision.^7^ This view has also been embraced by Xi, who has pledged technological independence in key areas: “Only by holding these technologies in our own hands can we ensure economic security, national security and security in other areas.” China’s 14th Five-Year Plan describes technological development as a matter of national security, not just economic development, marking a departure from previous plans.^8^ Western sanctions against Russia after the latter’s invasion of Ukraine seem to have only confirmed these views about the need for China to protect itself from the potential weaponization of interdependence (Asia 2022).^9^

The Chinese government has also embraced the importance of a dual circulation strategy, which aims to reduce China’s vulnerability to external shocks by increasing domestic consumption and reducing reliance on export-led growth.

### Left-wing and corporate power concerns: the need for common prosperity

While there is much to be celebrated about China’s economic advances, danger lurks in the country’s growing inequality and rising corporate power. New Left and neo-Maoist groups in China have objected to the country’s market transformation, framing the World Trade Organization as the tool of a “‘soft war’ waged by Western powers, particularly the United States and the United Kingdom, to pry open China’s markets for the benefit of Western corporations” (Blanchette 2019). These views, although present, have not been mainstream in Chinese debates, perhaps partly because they have not been endorsed by Xi and the Chinese Communist Party. What has taken center stage in Chinese government policy in recent years, however, are efforts to curb inequality and corporate power in the name of realizing “common prosperity.” Achieving a fair distribution of the gains from globalization, and not just growth per se, has become a critical goal.

While the mantra during the Deng Xiaoping years was to “let some get rich first,” Xi now emphasizes the need to focus on common prosperity: raising the fortunes of low-income groups, promoting fairness in society, making regional development more balanced, and emphasizing the importance of people-centered growth (Xi 2021). Whether it is cracking down on the private education sector or bringing China’s technology giants to heel, the narrative of common prosperity calls for a Chinese society that is more egalitarian and less liable to the economic inequalities among classes and regions that have led to populist backlash in many Western countries, most notably the United States. Hypercompetition embodied in the 9–9–6 culture, which has caused some young people to rebel by “lying flat,” is out; more equitable and sustainable approaches are in.

Although this narrative has a lot in common with the left-wing and corporate power narratives in the West, it is also mixed with some conservative cultural impulses—such as a desire to crack down on celebrities and “sissy boys”—that are more reminiscent of right-wing perspectives in the West. This narrative is not just critical of Wall Street-style greed, but also of Hollywood-style moral corruption.

On the whole, common prosperity is not viewed as being in tension with a commitment to economic globalization, which may reflect the fact that all income brackets in China gained over the last few decades, even if some gained more than others. The common prosperity push is in keeping with the Party’s professed socialist values, with equality being seen as an important tempering force for some of the excesses of capitalism.

### The interplay between narratives in the West and in China

What makes economic globalization complex is not only that it has many facets and looks different from different vantage points, but that its evolution depends on the (often unpredictable) interactions among many actors, including governments, companies, civil society, and individuals. The narratives about economic globalization circulating in the West and in China differ based on each side’s concerns and imperatives. But these domestic spheres are not isolated from each other. What is said and done within one group or country may be listened to and have effects in the other.

In some areas, the narratives in the West and China are mirror images of each other: the developments lamented by right-wing populists in the United States—in particular the movement of manufacturing jobs to Asia—are perceived as positive from the perspective of China and other Asian countries.

In other areas, the narratives clash with and fuel each other. The best example is the interaction of the Western geoeconomic narrative and the Chinese anti-hegemony narrative. In the West, concerns about a rising China have led to the increased securitization of debates about economic interdependence and to policies such as bans on 5G equipment manufactured by Huawei and export restrictions to curtail access by Chinese companies to advanced semiconductors. In China, these actions are perceived as examples of Western hegemony and as attempts to contain China’s rise, which in turn prompt the increased securitization of economic policies, in particular attempts to achieve technological self-reliance. This dynamic results in an amplifying feedback loop that has made geoeconomic framings ascendant in both China and the West in recent years, leading to increased pressures to decouple.

In yet other areas, Chinese and Western narratives seem to be running in parallel without much mutual reinforcement and amplification. Rising concerns about inequality and corporate power in both the West and China are giving rise to crackdowns on Big Tech on both sides through regulatory actions such as antitrust enforcement. In some ways, what is happening in one market could be seen as justification for the adoption of similar policies in the other market. For the most part, however, it seems that each debate is developing in a relatively autonomous way. The impetus for crackdowns in the United States appears to spring from domestic economic pressures, as well as a renewed appreciation of the importance of rigorous antitrust enforcement in the platform economy. And while President Xi seems to share concerns about the excessive power of platform firms, he is not justifying China’s crackdowns on the basis that the United States has engaged in similar actions.

Finally, on some global challenges, the two sides are largely in agreement. Both China and the West recognize the threats posed by the climate crisis and global pandemics. Even though they may disagree on the best strategies to adopt in response and on how to distribute the burden, these challenges provide opportunities for collaboration. Yet this insight is not easy to reconcile with the more conflictual narratives that are increasingly gaining hold on both sides. Ahead of the United Nations Climate Change Conference in 2021, US Climate Envoy John Kerry tried to frame the climate crisis through the lens of the global threats narrative instead of the geoeconomic one when he declared that “climate is not ideological. It’s not partisan, it’s not a geostrategic weapon or tool. … It’s a global, not bilateral, challenge” (Buckley and Friedman 2021). Yet Chinese Foreign Minister Wang Yi responded that cooperation on climate could not be separated from the overall environment of US–China relations. “The United States hopes to transform cooperation into an ‘oasis’ in Sino-US relations,” he said, “but if the ‘oasis’ is surrounded by ‘deserts,’ the ‘oasis’ will sooner or later be deserted” (Lelyveld 2021).

The United States is not the only side that is trying to manage the tension between different narratives. Following Russia’s invasion of Ukraine, China has attempted to avoid taking sides. Instead, it has tried to balance its relationship with Russia (in which the two are brothers-in-arms and friends-without-limits in the narrative against Western hegemony) with its desire for continued economic integration with the West (as per the win–win establishment narrative) while building its own resilience and pursuing targeted decoupling (as per the geoeconomic and against Western hegemony narratives). China recognizes that it has much to gain from a continuation of the establishment approach to economic interdependence, provided that the rules are interpreted broadly enough to allow China to maintain its own development model. We can also see companies attempting to balance their economic interest in remaining in certain markets with growing concerns about how geopolitical tensions and value conflicts might require them to exit those markets suddenly, as hundreds of Western companies did after Russia’s invasion of Ukraine.

## Conclusion

In recent years, events such as the US–China trade war, the COVID-19 pandemic, and Russia’s invasion of Ukraine have prompted commentators to proclaim the “end of globalization.” While these pronouncements are often made more for rhetorical flourish than for literal truth, they reflect an important phenomenon, namely, the fact that views about globalization—as captured in competing narratives—have been undergoing more profound change in recent years than in any other period since the end of the Cold War, when the economic establishment’s win–win narrative became preeminent.

As a result, globalization is not ending, but it is certainly changing. Responding to the populist backlash against globalization and motivated by growing security and environmental concerns, Western governments are seeking to strike a new balance between the efficiency gains that can be achieved through trade and investment liberalization, on the one hand, and the values of equality, security, resilience, and sustainability, on the other hand. The Chinese government is navigating very similar challenges, all while trying to find its footing as a leading economic and geopolitical power amidst growing skepticism and hostility from the West.

The relationship between China and the West will play a central role in shaping the future of globalization, but the relationship is highly complex, and its future development is deeply uncertain: possible future scenarios range from a political rapprochement and stronger cooperation on issues of common concern to radical economic and technological decoupling and even potential military confrontation. Understanding the main narratives about economic globalization in the West and China is a good way of starting to understand this complexity and beginning to think through how different narratives might intersect and interact in shaping the future of international economic relations.

As we argue in *Six Faces of Globalization*, when dealing with complex and contested issues, it is crucial to explore *multiple* perspectives. No single narrative can capture the multifaceted nature of such issues, and no perspective is neutral. Each narrative distills a certain set of experiences and tells part of the story; none tells the whole. Every narrative embodies value judgements about what merits our attention and how we should evaluate what we see; none is value free. You do not have to agree with every narrative—you may consider some to be wrong, empirically or normatively. But considering multiple narratives in a structured way helps everyone to be conscious of how their approach fits within the broader context and what they might be missing about what others are seeing and valuing.

Psychologists have recognized that complicating narratives by seeking to understand complex situations from multiple perspectives can help to move us past our tendency toward binary thinking and polarized disputes (Grant 2021; Coleman 2021; see also Ripley 2021). As relations between the West and China become more highly charged, and as the global challenges we all face become more pressing, the multi-perspective approach that we adopt and advocate for here becomes ever more important.

## Footnotes

1 In studies of peace and conflict, psychologists have found that leaders who demonstrate low levels of integrative complexity (i.e., the ability to see complex issues from multiple perspectives and integrate these into a more coherent whole) are less likely than their peers to produce negotiated outcomes and more likely to oversee violent eruptions. By contrast, leaders who are better able to understand the perspectives and priorities of different sides are more likely to find trade-offs and creative solutions that meet each side’s core concerns. See: Guttieri et al. (1995), Suedfeld and Tetlock (1977), Suedfeld et al. (1977).

2 For a similar theoretical approach, see Halliday and Shaffer (2015).

3 For the flying geese paradigm of development, see Akamatsu (1962). On China as the flying dragon, see Lin (2011).

4 For commentators on the “China threat theory”, see Xiang (2019), Liu (2018), Shi (2012), Lu (2013), Hu (2018), Xu (2019) and Yu (2018).

5 On big security, see Hu (2016) and People’s Daily (2019).

6 On self-reliance and mastering core technologies, Wang and Zhou (2018),CRI Online (2018).

7 See, for example, Yao (2019); Zeng (2019). See also Gewirtz (2020).

8 For Xi’s quote, see Xinhua (2021).

9 Including a discussion of a report prepared by China’s Ministry of Public Security and Ministry of State Security that analyzes what would happen if the US, Europe, and Japan were to react to a Taiwan emergency by imposing economic sanctions on China like they did against Russia over its invasion of Ukraine. This observation was also presciently made by Taisu Zhang on Twitter: “I think it’s pretty obvious Western countries are (partially) treating the current conflict and ensuing sanctions as a trial run for measures they might take against China in the future…. I mean, they’d be crazy not to, and Beijing would be crazy to not perceive it as such.” See Zhang (2022).
